# Tumour virology in the era of high-throughput genomics

**DOI:** 10.1098/rstb.2016.0265

**Published:** 2017-09-11

**Authors:** Ka-Wei Tang, Erik Larsson

**Affiliations:** 1 Department of Infectious Diseases, Institute of Biomedicine, The Sahlgrenska Academy, University of Gothenburg, Medicinaregatan 9A, 405 30 Gothenburg, Sweden; 2 Department of Medical Biochemistry and Cell Biology, Institute of Biomedicine, The Sahlgrenska Academy, University of Gothenburg, Medicinaregatan 9A, 405 30 Gothenburg, Sweden

**Keywords:** tumour virus, next-generation sequencing, virus integration

## Abstract

With the advent of massively parallel sequencing, oncogenic viruses in tumours can now be detected in an unbiased and comprehensive manner. Additionally, new viruses or strains can be discovered based on sequence similarity with known viruses. Using this approach, the causative agent for Merkel cell carcinoma was identified. Subsequent studies using data from large collections of tumours have confirmed models built during decades of hypothesis-driven and low-throughput research, and a more detailed and comprehensive description of virus–tumour associations have emerged. Notably, large cohorts and high sequencing depth, in combination with newly developed bioinformatical techniques, have made it possible to rule out several suggested virus–tumour associations with a high degree of confidence. In this review we discuss possibilities, limitations and insights gained from using massively parallel sequencing to characterize tumours with viral content, with emphasis on detection of viral sequences and genomic integration events.

This article is part of the themed issue ‘Human oncogenic viruses’.

## 1. Introduction

Seven known human tumour viruses, discovered using a variety of techniques, are causative agents for a large fraction of human cancers [1]. Animal and human model tumour viruses have served as important tools for studies of tumorigenesis and were essential in establishing key concepts such as oncogenes and tumour suppressor genes [2,3]. Recently, the introduction of massively parallel sequencing, also known as next-generation sequencing (NGS), has revolutionized characterization of genomic and transcriptomic alterations in tumours. In addition, NGS-based approaches are now increasingly being applied to the study of viral nucleic acids in tumours.

The most recently discovered human tumour virus, Merkel cell polyomavirus (MCV), responsible for the majority of Merkel cell carcinomas, was identified using a pioneering bioinformatical method, digital transcriptome subtraction of sequences generated by NGS [4]. The principle was reminiscent of earlier molecular biological techniques for enrichment and sequencing of viral genetic material, which led to the discovery of hepatitis C virus (HCV) and Kaposi's sarcoma associated herpesvirus (KSHV or human herpesvirus 8 (HHV8)) [5,6]. However, the larger amounts of data generated by NGS now allowed the enrichment process to be performed *in silico* using bioinformatics, by first removing human sequences followed by unbiased detection of viral traces in the remaining data. Variants of this approach have since been used in many subsequent NGS-based studies. These have confirmed previously described virus–tumour associations and added several other insights, including rare associations, novel recurrent sites of virus integration, and rejection of previously proposed associations. Here we review some of the contributions NGS has made in this field, with the main focus on detection of viral sequences in cancer.

## 2. Detection of viruses in tumours using high-throughput sequencing

NGS-based characterization of viral sequences in tumour material generally presents few experimental challenges, since standard protocols for nucleic acid extraction and sequencing are applicable. Sequencing libraries generated for general transcriptomic or genomic analyses of tumours can therefore be repurposed for viral analyses, which has enabled large cancer cohorts to be screened for viral content solely using bioinformatics and publicly available sequencing data [7–9].

**Figure 1.**
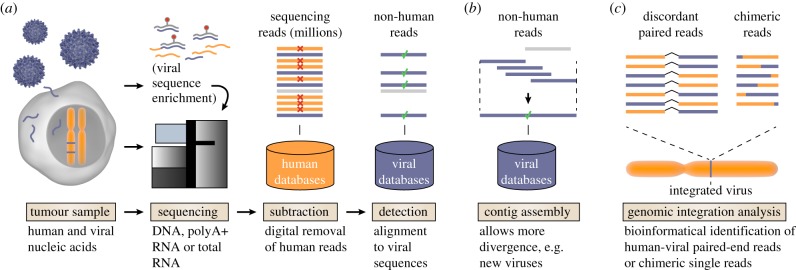
Detecting viruses in tumour samples using high-throughput sequencing. (*a*) RNA (polyA+ or total) or DNA prepared from tumour tissue using standard protocols is subjected to high-throughput sequencing, producing millions to billions of short sequencing reads. Alternative protocols allow for enrichment of viral nucleic acids prior to sequencing. Typically, human sequencing reads are then bioinformatically subtracted and the remaining data are compared against known viral reference sequences, such as available complete viral genomes. (*b*) Bioinformatical assembly of non-human reads into longer sequences (contigs) prior to comparison to viral references allows for detection of more distant evolutionary relationships including new viral species. (*c*) Viral genomic integrations can be revealed by identifying discordant read pairs from paired-end sequencing where one mate aligns to human and the other to viral reference sequences. Individual chimeric human-viral reads allow fine-mapping of genomic integration breakpoints.

Typically, tumour tissue is flash frozen after harvesting, and pathological tissue slides are prepared and analysed to define the borders of the tumour. Samples with high tumour content are then lysed and nucleic acids are purified. The type of nucleic acid analyte analysed (typically mRNA, total RNA or DNA) will determine what types of viruses can be detected and what kinds of studies may be performed. For example, DNA-based analyses can reveal integrated and latent non-expressed viruses, and may enable quantification of absolute viral load per human cell [10]. Transcriptome sequencing, on the other hand, may reveal non-retrotranscribed RNA viruses that will go undetected in DNA data, and also provides crucial insight into viral and host gene activities. Sequencing libraries are typically prepared by fragmentation into appropriate sizes and by adding specific adaptors to the fragments. Next, the fragments are immobilized to two-dimensional surfaces on flowcells, where they are amplified by solid phase PCR and subjected to a sequencing-by-synthesis reaction using fluorescently labelled nucleotides. With current technology, in the order of tens to hundreds of millions of short sequencing reads will be produced for a single transcriptome, and more still in the case of whole genome sequencing (WGS) (figure 1*a*).

The bioinformatical analysis generally involves removal of low-fidelity reads followed by matching against human reference sequences. Remaining non-human reads are finally matched against a viral genome database (figure 1*a*) [7–9,11–19]. Variability in sequencing depth is typically accounted for by normalizing to the total number of obtained reads, for example by stating viral expression levels as ‘reads per kilobase and million base pairs sequenced’ (RPKM) or in parts-per-million (ppm) of total library reads. Greater sensitivity for detecting highly diverged viral strains or new viruses can be obtained by first assembling non-human sequences into longer contiguous segments (contigs), followed by searches for homology to known viral reference sequences (figure 1*b*) [7,9,12,13,15,16,18]. Furthermore, sites of viral genomic integration can be bioinformatically pinpointed by identification of discordant paired reads or chimeric human-viral sequences (figure 1*c*, discussed below). Several software packages are now available to simplify these tasks, reducing the expertise required [7,14,17,18].

## 3. Reference results from known virus-associated tumours

Early NGS-based studies of tumour viruses were limited by the relatively low sequencing depth available at the time. Bioinformatical processing was carried out meticulously with every sequence read analysed and categorized [19]. Transcriptomic sequencing of four Merkel cell carcinomas using pyrosequencing yielded less than four-hundred thousand high-fidelity reads of which two unknown transcripts led to the discovery of the Merkel cell polyomavirus [4]. Later analyses of larger patient cohorts using more current sequencing methodologies have established NGS as an efficient method for detection of viral mRNA [8,9,11,20–23]. In particular, studies of tumours with known viral aetiology have been important in establishing a point of reference against which novel observations can be compared. With very few exceptions, these studies show that a single type of virus is predominant in each tumour.

Analysis of 85 cervical squamous cell carcinomas and endocervical adenocarcinomas, and 43 head and neck squamous cell carcinomas, infected with various types of high-risk human papillomaviruses (HPV), revealed an average of approximately 200 ppm of viral mRNA reads in relation to the total library size, ranging from 11 to 598 ppm for cervical squamous cell carcinoma and endocervical adenocarcinoma, and 22 to 848 ppm for head and neck squamous cell carcinoma. Similarly, the average hepatitis B virus (HBV) mRNA content in 11 liver hepatocellular carcinomas was also nearly 200 ppm, ranging from 2 to 854 ppm with three tumours containing less than 10 ppm viral mRNA [9]. The low proportion of HBV reads in some samples likely reflects the fact that some HBV-initiated liver tumours are able to proliferate in the absence of the viral genome.

Results from six AIDS-associated lymphomas containing Epstein–Barr virus (EBV/HHV4) revealed viral expression ranging from 145 to 8857 ppm, with an average of 2750 ppm EBV transcripts [24]. Interestingly, 24 EBV-positive gastric adenocarcinoma showed on average only 88 ppm viral mRNA, ranging from 4 to 300 ppm [22]. The significantly higher values in the AIDS-associated lymphomas may conceivably reflect the absence of humoral or cell-mediated surveillance.

A limit of 10 ppm viral mRNA reads (corresponding to 100 viral reads at a sequencing depth of 10 million reads) has been suggested as an approximate divider for tumours with clonal presence of expressed viruses, since most virally induced tumours were found to surpass this level [9]. Lower levels can however not always be disregarded, exemplified by the Merkel cell polyomavirus which was discovered at 5 ppm [4]. Additionally, results presented above mostly derive from studies using polyA enrichment prior to sequencing, which theoretically excludes certain viruses such as HCV of the Flavivirus family. Nevertheless, HCV sequences have still been detected at very low levels in polyA enriched libraries [9,11], and there may thus be technical reasons as to why even weaker signals should be considered in some cases.

Recent studies employing small RNA sequencing, whole exome sequencing (WXS) and WGS data for identification of viruses in tumours have produced results that are largely consistent with transcriptomic analyses [10,21–23,25]. It should be noted that the viral signals seen in WXS-based analyses are typically weak, sometimes with only single viral reads observed even in known virus-associated tumours [22], which is explained by the host sequence enrichment step inherent to the methodology. WGS, in contrast to WXS and transcriptome sequencing, produces a constant host genome background that can be useful to estimate absolute viral genome copy number per cell [10]. As an example, at one EBV (170 kb genome) per cell (6.4 Mb diploid genome), and assuming 100% tumour cell content, one would expect approximately 27 ppm of total library reads to be of viral origin.

## 4. Low-level detection and contamination

The sensitivity and unbiased nature of NGS gives rise to a new type of problem, where trace amounts of human as well as non-human viruses are often detected in tumours and control tissues [26–28]. These signals can arise for several reasons, one being infiltration of virus-positive lymphocytes in tumour tissue. This has been shown, for example, in AIDS-associated lymphomas, where low levels of EBV transcripts detected by NGS were confirmed by *in situ* hybridization to be due to infiltration of latently EBV-infected lymphocytes [24]. Beta (HHV5/CMV, HHV6 and HHV7) and gammaherpesviruses (HHV4 and HHV8) as well as HIV-1 are also known to infect and establish latency in haematopoietic progenitor cells and lymphocytes [29]. This likely explains why low levels of viral transcripts from these agents have been detected by NGS in tumours as well as healthy control tissue from several cancers [9,11,25].

Viral signals may also arise from infected tissue surrounding the tumour. Primary herpes simplex virus 1 (HSV1) infection occurs predominantly in the oropharyngeal area with ensuing cold sores [29]. Possibly, this explains high levels of HSV1 detected in one head and neck squamous cell carcinoma, which could not be confirmed by immunohistochemistry [21]. HSV1 has also been detected in several oesophageal carcinomas [11], but it is not clear whether these signals originate from tumour cells or surrounding cells.

An additional challenge is contaminants, which may be introduced at all steps during sample preparation or downstream processing [30]. Silica membranes in some nucleic acid extraction kits have been shown to contain algae viruses, which were mistakenly classified as a new hepatitis virus [31,32]. Other reagents and components of the laboratory environment can also contain contaminants, which may be of human, animal, invertebrate, plant, fungi and bacterial origin [26,27,33,34]. Unexpected microbial detections in NGS libraries have sometimes been linked to specific sequencing centres and timepoints, further supporting that they represent contaminants [34,35].

Sequences from non-human viruses are typically present only in low amounts when detected by NGS in tumours [9,26]. Although possibly explained by zoonotic or environmental infections, most of these signals likely arise from contamination during sample processing or sequencing, or environmental exposure at the tumour site. The association of the murine XMRV with human prostate cancer mislead the scientific community for many years. Thorough investigation, including reanalysis of the original tissue sample, finally revealed this to be due to contamination [36]. Several NGS-based studies have since confirmed the absence of XMRV in large prostate cancer cohorts [8,9,11,13,37,38].

A frequently found synthetic viral contaminant is the immediate early promoter of the human cytomegalovirus (HHV5 or CMV) used in many mammalian expression plasmids [9–11,25]. Additionally, intentional phage spiking of sequencing libraries may cause confusion during downstream analyses [39]. Another possible source of synthetic viral sequences are cell lines where viral agents such as HPV, adenoviruses, EBV, retroviruses and SV40 have been used for transformation [12]. HeLa cells naturally harbour HPV18 and have been known to cross-contaminate cell lines throughout the world. Recently, it was shown that low levels of HPV18 detected in some colorectal tumours sequenced by The Cancer Genome Atlas (TCGA) were due to HeLa contamination, as evidenced by an identical HeLa-specific HPV genotype in these samples [35]. The cutaneous HPV38 has also been suggested to be present as a contaminant in endometrial cancer RNA sequencing libraries from TCGA [40]. Also in TCGA data, a single clear cell renal cell carcinoma was found to contain HBV mRNA [9]. However, closer analysis also revealed weak but consistent expression of liver marker mRNAs, supporting contamination by HBV-positive liver tumour mRNA.

The examples described here stress the need to maintain a critical standpoint towards novel virus–tumour associations detected by NGS. Negative control samples and complementary laboratory assays such as *in situ* hybridization, immunohistochemistry and PCR of tumour and healthy tissue are useful to confirm initial findings [4,21,24,41]. Additionally, lack of viral genetic diversity in between different samples may indicate presence of a common contaminant [35,42]. Finally, even confirmed presence of a virus naturally does not imply causation, and overlapping epidemiologies for different viruses may further complicate interpretation. Conversely, viral presence is not obligate in tumours initiated by chronic inflammation caused by viruses. Specific criteria for defining virus–tumour associations are therefore discouraged, and we should not rely solely on one method but find multiple biological indicators that together convincingly can justify the virus as a causative agent [43].

## 5. Rare virus–tumour associations

Viruses that have co-evolved with humans as their main host are typically highly selective in terms of the cell types they can infect [44]. Detections beyond this preferred range of cell types are therefore uncommon and can indeed often be explained by contaminations, as discussed above. However, some rare associations detected by NGS warrant further consideration.

HPV16 is one of the few viruses that have been associated with tumours outside the primary sites of infection in the ano-genital region. In addition to expected detections in head and neck and cervical tumours, HPV16 has been found in single uterus, lung and bladder carcinomas sequenced by TCGA [8,9,11]. While supported by some earlier studies [45–47], this still represents a very small fraction of tumours from these locations. Additionally, a recent NGS-based study reported HPV16 in 3 out of 530 low grade gliomas [11,25]. Further verification using *in situ* hybridization or immunohistochemistry is needed to confirm these observations.

EBV is another agent implicated in a wide range of cancers including Burkitt's lymphoma, nasopharyngeal carcinoma, Hodgkin's lymphoma and gastric adenocarcinoma. Additionally, transcriptomic analysis recently revealed high levels of EBV in 2 out of 105 diffuse large B-cell lymphomas (432 and 37 ppm) [48]. Two tumours were also positive for HHV6 in this cohort (99 and 19 ppm), in one case coinciding with EBV infection. Analysis of viral gene expression patterns further supported a causal role for EBV, while HHV6 was suggested to be due to disease-related immunosuppression.

A single bladder urothelial carcinoma, out of 316 characterized using transcriptome sequencing by TCGA, was found to contain BK polyomavirus (BKV) [8,9,20]. Earlier reports of this virus in bladder carcinoma using low-throughput diagnostic methods have been contrasting [49]. The oncogenic BKV T-antigen was expressed at high levels (318 ppm) and the viral genome was shown to be integrated into the host genome [9]. Although this supports a functional contribution from BKV in rare cases, it should be noted that BK as well as other polyomaviruses often cause asymptomatic infections and are ubiquitous in humans [50].

## 6. Non-detection

The literature contains a large number of proposed virus–tumour associations that are controversial. These are typically based on traditional viral diagnostic techniques, including PCR, immunohistochemistry, *in situ* hybridization and western blotting, all of which are prone to false positive detections. Modern genomic approaches, which allow unbiased screening of large tumour cohorts, have the potential to bring clarity to some of these proposed associations.

During the 1960s and 70s, it was widely believed that HSV2, which causes genital herpes, was the causative agent for cervical carcinoma. High-risk HPV types were later identified in these tumours and years of disputes followed [51] before high-risk HPV was finally established as the *de facto* causative agent [52]. Today, NGS-based studies of large cohorts confirm that more than 90% of cervical carcinomas express high levels of high-risk HPV, while no HSV2 sequences can be detected [9,11,23]. Hence, this could have been clarified faster had high-throughput sequencing been available at the time.

Breast cancer is the most common invasive cancer in the world, and has been extensively studied. Several viruses including EBV, HPV and MMTV (mouse mammary tumour virus) have been implicated [53]. Frequent clonal presence and expression of EBV or HPV can be ruled out, considering that transcriptomic data from more than 800 breast tumours have now been analysed without any significant levels of these viruses being detected [8,9,54]. A small number of reads aligning to MMTV (9 out of more than 1.5 billion) were detected in the same cohort [9]. These trace amounts are suggestive of contamination, but silent genomic integration could still be possible and WGS-based analysis is warranted to resolve this.

The role of CMV in human cancer has been highly controversial. After the initial claim that CMV DNA and protein was found clonally in the majority of gliomas [55], CMV has now been associated with a wide variety of other cancers in the literature. Ubiquitous presence of CMV has been proposed in most types of brain tumours, but this has been contested in other reports [56]. All NGS-based studies of non-enriched glioma material, in total more than 700 samples, have concluded that CMV RNA cannot be detected [8,9,11,16,25]. Likewise, analysis of deep coverage WGS data from 34 glioblastoma multiforme tumours failed to reveal CMV [10]. The examples discussed here show that NGS-based approaches have great potential to bring clarity to debated virus–tumour associations.

## 7. Viral genomic integration

Retroviruses such as HTLV-1 (human T-lymphotropic virus type 1) establish chronic infection by genome integration, which causes the virus to be propagated in the host for long periods of time [57,58]. Integration of DNA viruses into tumour genomes appear to be random events, although these may be facilitated by disruption of DNA repair pathways by viral gene products [59,60]. The integration of MCV appears to be obligate for tumorigenesis of Merkel cell carcinoma, while certain types of HPV display a low rate of integration [9,11,61]. Genomic viral integration may contribute to cellular transformation by insertion of strong viral promoters near oncogenes or by disruption of tumour suppressors [60,62]. By using high-throughput sequencing, it is now possible to study these events in great detail on a genome-wide scale.

Two main principles, applicable to both DNA and RNA sequencing data, are used for detection of viral integrations (figure 1*c*) [7–9,11,13,14,16,17,40,63–65]. The first involves identification of discordant viral-human read pairs in paired-end sequencing data, where both tails of DNA fragments are sequenced. Challenges include false chimeric pairs that may arise for technical reasons [9]. The second approach takes advantage of individual reads that map partly to human and partly to viral sequences. This offers base-pair resolution, but such reads are more rare and higher sequencing depth is therefore required. Performance can be improved by applying a combination of both aforementioned techniques, by enrichment of viral sequences prior to sequencing (figure 1*a*), and by combining transcriptome and WGS data [62,66].

HPV integrations have been studied for decades, and methods for enrichment and sequencing of integration points have been developed both for fusion transcripts and integrated DNA [60]. Recent studies using NGS have verified previously described integration breakpoint hotspots in both the viral and host sequence [11,67]. Several studies have shown that integrations often coincide with copy number gains and cellular genes showing elevated expression [9,11,21,23]. Possibly, initiation of replication of integrated viral DNA and subsequent activation of DNA damage responses can contribute to copy number gains in these regions [68].

Likewise, HBV integration has been thoroughly studied, and recurrent viral integrations have been detected in specific genomic regions [69]. During primary HBV infection the virus spreads throughout the whole liver. Interestingly, NGS-based analyses suggest that the preferred locations for integration may differ between tumours and surrounding liver, since integrations in the *FN1* gene were frequent only in non-cancerous tissue [11,69–71].

EBV integration has previously been described in cell lines, but not in tumours. Surprisingly, transcriptomic analysis of 24 EBV-positive gastric adenocarcinomas revealed several reads that supported integration in one single tumour, suggesting that EBV integration may occur also in clinical samples [22].

Comprehensive mapping of HTLV-1 integrations by enrichment of insertion sites before sequencing has enabled monitoring of latently infected CD4+ T-lymphocyte populations before the onset of adult T-cell leukaemia, and has revealed that malignant cells most often originate from single clones and not through oligoclonal proliferation as previously proposed [57,65]. As exemplified here, NGS-based approaches have been useful for confirming as well as extending our knowledge of viral integrations in tumours genomes.

## 8. Future perspectives

NGS was successfully used in the discovery of MCV in Merkel cell carcinoma [4], but it should be noted that subsequent attempts to identify novel oncogenic viruses in large patient cohorts using genomic approaches have been unsuccessful [8,9,11]. However, current *in silico* methods are highly dependent on existing databases of viral sequences, and many new viruses, including potential human pathogens [72,73], have been uncovered in recent years. While unlikely, it is possible that a new, sequence-unique, family of tumour viruses awaits discovery, possibly in rare tumours or in immunosuppressed patients, and it is almost certain that NGS would play a key role in such a finding.

Sequencing technologies continue to improve at a rapid pace. Each new generation of machines promises longer reads, shorter run times, and lower per-base-pair cost. While current technology is more than sufficient for the detection of clonally present viruses in tumours, many benefits can still be expected as the amount of data continues to increase. For example, more widespread use of total RNA transcriptomic analyses, rather than the polyA enriched protocols that dominate today, will provide a more comprehensive description of cellular RNA content including non-polyA viruses. High-coverage WGS, which is becoming increasingly accessible, gives improved possibilities for mapping of viral integration breakpoints, quantifying viral load, and better statistical strength for rejecting controversial virus–tumour associations. Larger cohorts will increase the chances of finding new viruses or rare associations. Furthermore, single-cell sequencing approaches will be able to shed light on heterogeneity in cell populations, including the interplay between virus-infected and surrounding cells [74].

The discovery of human tumour viruses has had a profound effect on the prevention of human cancer. Efficient vaccines and antiviral treatment have completely shifted the aetiological causes for cancer in developed countries. It is therefore of great importance for the scientific community to remain vigilant in the search for new virus–tumour associations. NGS is now proven to be extremely efficient for characterization of tumour viral content, and will soon be the primary tool for discovering, confirming, as well as rejecting virus–tumour associations.

## Data accessibility

This article has no additional data.
